# Phase I and pharmacokinetic study of XR11576, an oral topoisomerase I and II inhibitor, administered on days 1–5 of a 3-weekly cycle in patients with advanced solid tumours

**DOI:** 10.1038/sj.bjc.6602178

**Published:** 2004-09-28

**Authors:** M J A de Jonge, S Kaye, J Verweij, C Brock, S Reade, M Scurr, L van Doorn, C Verheij, W Loos, C Brindley, P Mistry, M Cooper, I Judson

**Affiliations:** 1 Department of Medical Oncology, Erasmus Medical Center/Daniel den Hoed Cancer Center, Groene Hilledijk 301, 3075 EA Rotterdam, The Netherlands; 2 Royal Marsden Hospital, Fulham Road, London SW3 6JJ, UK; 3 Quintiles Limited, Research Avenue South, Heriot Watt University, Research Park, Riccarton, Edinburgh, EH14 4AP, UK; 4 Xenova Limited, 957 Buckingham Ave, Slough, SL1 4NL, UK; 5 Millennium Pharmaceuticals Inc, 350 Massachusetts Avenue, Cambridge, MA 02139, USA

**Keywords:** XR11576, topoisomerase I inhibitor, phase I, solid tumours, oral

## Abstract

XR11576 is an oral topoisomerase I and II inhibitor. The objectives of this phase I study were to assess the dose-limiting toxicities (DLTs), to determine the maximum tolerated dose (MTD) and to describe the pharmacokinetics (PKs) of XR11576 when administered orally on days 1–5 every 3 weeks to patients with advanced solid tumours. Patients were treated with escalating doses of XR11576 at doses ranging from 30 to 180 mg day^−1^. For PK analysis, plasma sampling was performed during the first and second courses of treatment and XR11576 concentrations were assayed using a validated high-performance liquid chromatographic assay with mass spectrometric detection. In all, 21 patients received a total of 47 courses. The MTD was reached at 180 mg day^−1^, with diarrhoea and fatigue as DLT. Nausea and vomiting, although not qualifying for DLT, was ubiquitous. Only in combination with an extensive prophylactic antiemetic regimen consisting of a combination of both dexamethasone and a 5HT3 antagonist was treatment with XR11576 at 120 mg day^−1^ tolerable. The systemic exposure of XR11576 increased more than proportionally with increasing dose, with a large interpatient variability. No objective responses were seen; four patients experienced stable disease for periods of 12–30 weeks. In this study, the DLTs of XR11576 were diarrhoea and fatigue. The recommended dose for phase II studies of XR11576 is 120 mg administered orally, on days 1–5 every 21 days. Alternative regimens are currently being explored.

DNA topoisomerases are essential nuclear enzymes involved in multiple nuclear functions such as chromosomal recombination, DNA repair, transcription and chromatin assembly (Eng *et al*, 1988; Hsiang *et al*, 1989). Topoisomerase I and II inhibitors are of great clinical interest because of their important antitumour activity in a broad spectrum of tumour types (Rothenberg, 1997). The expression of topoisomerase I and II*β* does not vary significantly during the cell cycle, whereas topoisomerase II*α* expression increases during the S phase and reaches a peak at G_2_/M phase (Heck *et al*, 1988). It has been shown in preclinical studies that crossresistance between topoisomerase I and II inhibitors is unusual in resistant cell lines (Ferguson *et al*, 1988). Alterations in the regulation of one topoisomerase are often compensated by alterations in the other (Lefevre *et al*, 1991). Joint inhibitors of topoisomerase I and II appear to combine the properties of the individual specific inhibitors but act across the cell cycle, and so target a larger population of cells in any asynchronous population, resulting in greater antitumour activity. Several of these ‘dual inhibitors’ have been identified, including intoplicin, saintopin, XR5000 and F11782 (Newman *et al*, 1999; Twelves *et al*, 1999; Etievant *et al*, 2000; Denny and Baguley, 2003).

4-methoxy-benzo[*a*]phenazine-11-carboxylic acid (2-dimethulamino-1-*R*-methyl-ethyl)-amide (XR11576) is a novel substituted benzo[*a*]phenazine-11-carboxamide that targets both topoisomerase I and II (Figure 1

**Figure 1:**
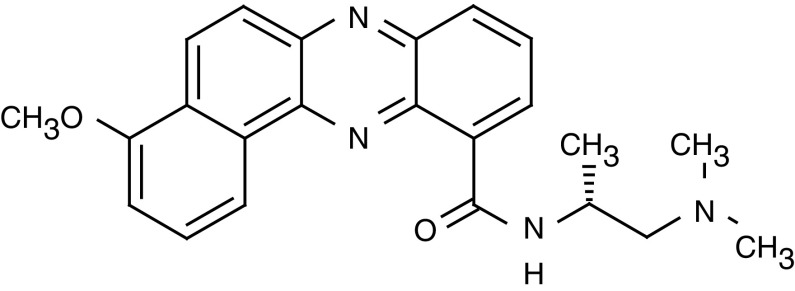
Chemical structure of XR11576.

) (Jobson *et al*, 2002; Wang *et al*, 2002).

*In vitro*, XR11576 exerted antitumour activity against a variety of murine and human tumour cell lines, including rapidly proliferating murine leukaemias and human colon, breast, ovarian and lung carcinoma cell lines. *In vivo*, XR11576 was highly active against human colon and small-cell lung cancer xenografts (Mistry *et al*, 2002; Lewis *et al*, 2003). XR11576 was also active against multidrug-resistant cells overexpressing P-gp or MRP, or against cells with downregulation of topoisomerase II levels (Di Nicolantonio *et al*, 2002). On giving XR11576 orally to rodents and dogs, its antitumour activity was preserved.

In animals, XR11576 predominantly induced bone marrow and gastrointestinal toxicity. In addition, when i.v. doses were repeatedly administered to dogs, myocarditis and nephritis were observed. However, these toxicities were not observed in studies of repeated oral dosing. Furthermore, no cardiac toxicity was detected in a cardiovascular safety study (data on file, FPD 1055). The maximum tolerated doses (MTDs) in a 14-day oral administration schedule in rats and 8/9-day oral administration schedule in dogs were 150 and 200 mg m^−2^, respectively. The oral route of administration was chosen because the i.v. route induced unacceptable vein irritation and because XR11576 had proven activity when given orally, which would enable a more convenient method of prolonged drug administration and create the opportunity for cost-effective outpatient therapy.

The pharmacokinetics (PKs) of XR11576 have been studied in several animal species. After single intravenous administration, XR11576 rapidly distributed from plasma into the tissues, resulting in a large volume of distribution. After both i.v. and oral administration to mice and rats, high tissue levels of XR11576 relative to plasma were observed, with the highest and most prolonged drug levels in tumour tissue. The oral availabilities of XR11576 in mice and rats were 72±25 and 54±32%, respectively. In mice, food reduced the bioavailability. After single oral administration to dogs, the systemic exposure increased more than dose-proportionally. The unchanged drug was the major component in plasma. However, there was evidence of hepatic metabolism to at least one major metabolite. Plasma half-life varied between 1 and 9 h. Repeated daily oral doses in dogs did result in a reduction in the systemic exposure of approximately 60%, which might be related to the observed gastrointestinal toxicity. The main route of elimination of XR11576 is likely to be hepatic metabolism.

The purposes of the present phase I study were to determine the MTD of XR11576 administered orally on days 1–5 every 21 days, to establish the dose-limiting and other toxic effects, to describe the PK of XR11576, to document any antitumour effects and to establish a dose suitable for further phase II evaluation of activity of the compound.

## PATIENTS AND METHODS

### Patient selection

Patients with a cytologically or histologically confirmed diagnosis of a malignant solid tumour refractory to standard forms of therapy were eligible for this study, provided that they met the following criteria: age between 18 and 75 years; WHO performance status ⩽1; estimated life expectancy ⩾3 months; no previous anticancer therapy for at least 4 weeks; no major fluid effusions; no significant gastric or small intestine disease that might influence the absorption of the drug; and adequate haematopoietic (haemoglobin ⩾5.2 mmol l^−1^, absolute peripheral granulocyte count ⩾2.0 × 10^9^ l^−1^ and platelet count ⩾100 × 10^9^ l^−1^), hepatic (bilirubin⩽the upper normal limit, and serum aspartate aminotransferase and alanine aminotransferase⩽3.0 times the upper normal limit) and renal (serum creatinine concentration⩽the upper normal limit) functions. Patients with symptomatic brain or leptomeningeal metastases were excluded. Patients had to be able to swallow size 1 capsules. All patients gave written informed consent before study entry. The study was approved by the Institutions' Medical Ethic Committees.

### Treatment and dose escalation

XR11576 was supplied by Fulcrum Pharma Developments Ltd (Hemel Hempstaed, UK) as size 1 gelatin capsules comprising 5.0, 20.0, 60.0 and 120.0 mg of active drug and dibasic calcium phosphate anhydrous. The capsules were stored at room temperature (25°C or below). Capsules were taken once a day, with a glass of water after an overnight fasting of at least 2 h before breakfast. The daily dose of XR11576 was provided in separate boxes, each of which was clearly identifiable by the patient. Patients were instructed to record their daily amount of capsules taken, the time of administration and the timing in relation to breakfast. Compliance with the scheduled treatment was assessed at the end of each course, by counting the used and returned capsules of XR11576 in relation to the record kept by the patient for the given cycle. With the exception of the first and second courses, during which patients were hospitalised for PK sampling, patients were treated on an outpatient basis.

The starting dose of XR11576 was 30 mg day^−1^, one out of three of the no observed adverse effect level and one out of 10 of the MTD in rat and dog. Since for most anticancer agents dosing according to body surface area does not reduce the interpatient variability in PKs, it was decided to use absolute doses of XR11576 and study the relation between oral clearance of XR11576 and the body surface area (Ratain, 1998). The total dose prescribed was rounded to the nearest 5 mg. At least three patients were entered at each dose level. Dose escalations between cohorts were based on the prior dose level's toxicity and pharmacological data, allowing a dose escalation up to 100% (which was determined by the worst significant toxicity). Once ⩾grade 2 toxicity was observed in one patient, further dose escalation did not exceed 50%. The stopping dose was defined as the dose level that induced dose-limiting toxicity (DLT) during course 1 in ⩾2/3 or ⩾2/6 patients. Dose-limiting toxicities were defined as grade 4 granulocytopenia for 7 or more days, febrile neutropenia, platelets <25.0 × 10^9^ l^−1^ and/or nonhaematological toxicity ⩾grade 3. Nausea and vomiting subsequently responding to antiemetic therapy were excluded from DLT. Treatment with XR11576 was resumed when the ANC count had recovered to ⩾2.0 × 10^9^ l^−1^, the platelet count was ⩾100 × 10^9^ l^−1^ and nonhaematological toxicity had recovered to their baseline values. If a patient encountered DLT, the dose of XR11576 was decreased by one dose level at re-treatment. In case the toxicity had not recovered within 2 weeks of the planned re-treatment time, the patient would go off-study. At the dose recommended for further studies, the number of patients treated could be expanded to 12. Toxicities were evaluated according to the National Cancer Institute Common Toxicity Criteria, version 2.0 (ctep.info.nih.gov/reporting/in dex.html).

### Treatment assessment

Before treatment, a complete medical history was recorded and a physical examination performed. A complete blood count (CBC) including white blood cell differential and serum biochemistry, which involved sodium, potassium, calcium, urea, creatinine, uric acid, total protein, albumin, total bilirubin, alkaline phosphatase, *γ*-glutamyl transferase, aspartate aminotransferase, alanine aminotransferase, lactate dehydrogenase and glucose, were performed, as were urinalysis, pregnancy test, relevant tumour markers, electrocardiogram and a chest X-ray. The electrocardiogram was also performed 2 and 24 h after the first drug administration in the first and second cycles. Weekly evaluations included history, physical examination, toxicity assessment according to the CTC criteria version 2.0, and serum chemistries. Complete blood count was determined twice weekly. Tumour evaluation was performed after every two courses according to the Response Evaluation Criteria in Solid Tumours (RECIST) (Therasse *et al*, 2000). Patients were taken off protocol at the onset of disease progression.

### Sample collection and drug analysis

For PK analysis, 58 blood samples (∼5 ml each) were obtained from an indwelling intravenous cannula and collected in vials containing lithium heparin as anticoagulant. The samples were taken immediately before dosing, 30 min and 1, 1.5, 2, 3, 4, 6, 8 and 10 h after administration of the drug on days 1 and 4 (or 5) of the first and second courses. Additional samples were taken immediately prior to dosing on days 2, 3, 4 and 5, and 24, 48, 72, 144 and 312 h after the dose administration on day 5, both in the first and second cycles. From the third cycle onwards, PK samples were taken prior to dosing on day 5 to measure the potential accumulation of XR11576.

All samples were centrifuged immediately after sampling at 3000 r.p.m. for 10 min at 4°C and the plasma was stored at −20°C or lower in polypropylene tubes in the dark until analysis. A total of four urine samples were also collected over a 24-h period; 0–4, 4–8, 8–12 and 12–24 h post-dosing on day 1 of the first cycle only. Of the total amount collected, a measured quantity of 10 ml was drawn off and stored at −80°C until analysis. Concentrations of XR11576 in plasma were determined according to a validated liquid chromatography-mass spectrometry detection method with liquid–liquid extraction at the Drug Metabolism and Pharmacokinetics Department of Quintiles Limited (Internal Report at Quintiles). The lower limit of quantitation was 5 ng ml^−1^.

### PK data analysis

The apparent terminal half-life (*T*_1/2_(*z*)) of XR11576 was calculated as ln 2/*k*, where *k* is the terminal elimination rate constant (expressed in h^−1^). The peak plasma concentrations (*C*_max_) and the time to peak plasma concentration (*T*_max_) were determined from the experimental values. The areas under the plasma concentration–time curve (AUC) of XR11576 were estimated using the experimental values (trapezoidal rule) with extrapolation to infinity (AUC_0–∞_) using the apparent terminal rate constant, defined as the slope of the final three to four data points of the log-linear concentration–time plot. The total body clearance (CL) was calculated as the ratio between the administered dose and the AUC_0–∞_. The extent of accumulation (*R*_0_) in plasma was calculated from AUC_0–24 h_ (days 4 or 5)/AUC_0–24 h_ (day 1). The fraction of the administered dose (*F*_e_) excreted in urine and the cumulative amount of XR11576 excreted in urine (*A*_e_) was determined. PK data analysis was carried out using a noncompartmental analysis approach with the aid of WinNonlin Version Pron4.0.1 (Pharsight, Cary NC 27511).

#### Statistical analysis

A nonlinear power model was used to assess dose proportionality separately for each day during cycles 1 and 2. Statistical analyses were performed using SAS version 8.1.

## RESULTS

Between January 2002 and April 2003, 22 patients, whose main characteristics are listed in Table 1

**Table 1:** Patient characteristics

**Characteristic**	**No. of patients**
No. entered	22
No. assessable	21
	
*Age (years)*	
Median 56	
Range 35–70	
	
*Sex*	
Female	13
Male	8
	
*Performance status*	
WHO 0	3
WHO 1	18
	
*Tumour type*	
Colorectal	6
Cervical	4
Melanoma	4
SCLC	2
Miscellaneous	5
	
*Previous treatment*	
Chemotherapy	9
Chemotherapy and radiation	11

, were enrolled onto the study at two centres. All patients were eligible. One patient, entered at the 120 mg day^−1^ dose level, did not receive any medication due to deterioration of his condition prior to study start. The majority of the patients were either asymptomatic or had only mild symptoms at study entry. Patients were pretreated with a median of two prior chemotherapy regimens (range 1–6). The total number of assessable courses was 47. The median number of courses per patient was 2 (range 1–10). Dose levels studied were 30, 60, 120, 180 and 150 mg day^−1^ XR11576 administered orally on days 1–5, with courses repeated once every 21 days. Toxicity did not necessitate dose reductions. However, three patients went off study because of the toxicity experienced at the 120 (1 patient) and 180 mg day^−1^ (2 patients) dose levels. In the absence of DLT, the dose of XR11576 was escalated from 30 to 180 mg day^−1^. Since all previously entered patients on the 120 mg day^−1^ dose level had experienced grade 2 nausea/vomiting during treatment, which could not be ameliorated by the use of prokinetic antiemetics, it was decided to introduce the use of prophylactic 5HT_3_ antagonists at the 180 mg day^−1^ dose level. At the 180 mg day^−1^ dose level, DLT was encountered in two of three patients. One of the patients experienced fatigue grade 3 in the first treatment course. The second patient considered to have DLT experienced in her first cycle grade 2 diarrhoea, vomiting and fatigue. For a coincident upper airway infection, she was treated with antibiotics. In her second cycle, she experienced diarrhoea grade 4 in combination with nausea grade 1, fatigue grade 2, hypotension grade 3 and electrolyte disturbances grade 3. Although the diarrhoea might have been aggravated by the previous use of antibiotics, the event was considered study drug related. In spite of the fact that the DLT in this second patient was observed in the second cycle, it was decided that the dose of 180 mg day^−1^ was not feasible. At this point, the previous dose level of 120 mg day^−1^ was expanded. The first two additional patients did not encounter DLT. However, despite the prophylactic use of 5HT_3_ antagonists, they experienced nausea/vomiting grade 2. Therefore, the next three patients at the 120 mg day^−1^ dose level received a combination of 5HT_3_ antagonists and dexamethasone prophylactically, rendering the level of nausea and vomiting acceptable. One of the additional patients at this dose level experienced diarrhoea grade 3. It was subsequently decided to explore an intermediate dose level: 150 mg day^−1^ in three patients. At this dose 1, patient experienced grade 3 diarrhoea accompanied by neutropenia grade 4, nausea/vomiting grade 2 and fatigue grade 2, considered to be DLT. All three patients showed nausea/vomiting grade 2 despite the use of prophylactic 5HT_3_ antagonists and dexamethasone prior to and during the 5 days of treatment administration, and despite the use of a tapering dose of dexamethasone in combination with metaclopramide during the 3 days following therapy. On the basis of these results, and in view of XR11576's development as an oral therapy, the 150 mg day^−1^ dose level was not considered feasible and the recommended dose of XR11576 for further studies is 120 mg day^−1^ administered on days 1–5 every 3 weeks.

### Tolerability

Diarrhoea and fatigue were the principal DLTs and were observed both at the 180 and 150 mg day^−1^ dose level. They also constituted the most common nonhaematological effects of XR11576 together with nausea and vomiting (Tables 2

**Table 2:** Toxicity in the first cycle according to NCI-CTC version 2.0

		**WBC**				**ANC**				**Plt**				**Nausea**			**Vomiting**				**Fatigue**				**Diarrhoea**				
**Dose (mg day^−1^)**	**No. pts per cycle**	**1**	**2**	**3**	**4**	**1**	**2**	**3**	**4**	**1**	**2**	**3**	**4**	**1**	**2**	**3**	**1**	**2**	**3**	**4**	**1**	**2**	**3**	**4**	**1**	**2**	**3**	**4**	**DLT**
30	3/13	–	–	–	–	–	–	–	–	–	–	–	–	2	–	–	–	–	–	–	1	1	–	–	–	–	–	–	–
60	4/10	–	–	–	–	–	–	–	–	–	–	–	–	3	–	–	2	–	–	–	1	–	1	–	1	–	–	–	–
120	8/16	1	–	–	–	–	–	–	–	–	–	–	–	5	3	–	1	2	2	–	3	3	–	–	1	2	1	–	1
180	3/5	–	–	–	–	–	–	1	–	2	–	–	–	3	–	–	–	3	–	–	1	1	1	–	1	1	–	–	1
150	3/5	–	1	–	–	–	–	–	1	1	–	–	–	1	2	–	–	3	–	–	–	3	–	–	1	1	1	–	1

No. pts=number of patients; Plt=platelets; DLT=dose-limiting toxicity.

and 3

**Table 3:** Worse toxicity per cycle for all cycles according to NCI-CTC version 2.0

		**WBC**				**ANC**				**Plt**				**Nausea**			**Vomiting**				**Diarrhoea**				**Fatigue**			
**Dose (mg day^−1^)**	**No. pts per cycle**	**1**	**2**	**3**	**4**	**1**	**2**	**3**	**4**	**1**	**2**	**3**	**4**	**1**	**2**	**3**	**1**	**2**	**3**	**4**	**1**	**2**	**3**	**4**	**1**	**2**	**3**	**4**
30	3/13	–	–	–	–	–	–	–	–	–	–	–	–	3	2	–	3	–	–	–	1	–	–	–	1	2	–	–
60	4/10	–	–	–	–	–	–	–	–	–	–	–	–	6	–	–	4	–	–	–	1	–	–	–	2	2	2	–
120	8/16	2	–	–	–	–	–	–	–	1	–	–	–	8	7	–	3	5	2	–	3	3	1	–	5	5	1	–
180	3/5	–	–	–	–	–	–	1	–	2	–	–	–	3	1	1	–	5	–	–	1	1	–	1	1	1	2	–
150	3/5	–	1	–	–	–	–	–	1	1	–	–	–	2	2	–	1	3	–	–	1	1	1	–	–	4	–	–

No. pts=number of patients; Plt: platelets.

). Both the incidence and severity of diarrhoea increased with the dose of XR11576 administered. The median day of onset was day 5 (range 1–15 days), with a median duration of 4 days (range, 1–14 days). In most patients, the diarrhoea was self-limiting. Five patients required treatment with loperamide. Patients treated at the lower dose levels did not routinely receive antiemetic premedication with their first dose. However, once established, nausea and vomiting were difficult to treat. Therefore, it was decided to introduce prophylactic treatment with 5HT_3_ antagonists at the 180 mg day^−1^ dose level. Since single-agent treatment with 5HT_3_ antagonists could not prevent nausea or vomiting, it was decided to combine the 5HT_3_ antagonists with dexamethasone for the additional patients treated at the 120 mg dose level. Although the treatment with XR11576 was better tolerated, all patients experienced mild to moderate nausea. Only one patient developed grade 1 alopecia during treatment with XR11576.

Overall, the haematological toxicity was mild (Tables 2 and 3). Only one patient, who was heavily pretreated, experienced grade 4 neutropenia, which was not complicated by fever. In the patient who received 10 cycles, there was no evidence of cumulative haematological toxicity. Thrombocytopenia was limited to grade 1 and 2, and only observed in three patients, two of whom were treated at the highest dose level. In seven patients grade 1 anaemia was encountered, in four patients grade 2 anaemia. Anaemia did not seem to be dose related.

Electrocardiograms were performed prior to treatment and following first drug administration. No significant abnormalities in ECG tracings were observed. No clinical signs of impaired cardiac function were observed in any of the patients treated.

No treatment-related deaths were reported.

### Antitumour activity

No objective responses were observed. Four patients experienced stabilisation of their disease (melanoma, cervical cancer and parotid cancer) for a median duration of 12 weeks (range 12–30 weeks).

### Pharmacokinetics

Full kinetic data were obtained on days 1 and 4 from 21 patients following the administration of XR11576.

**Figure 2:**
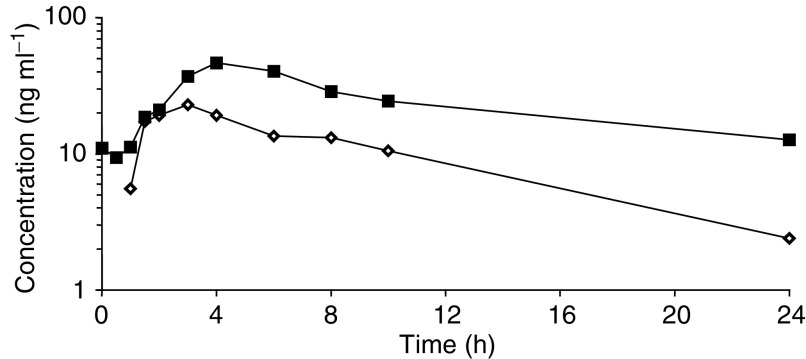
Representative plasma concentration–time profile of XR11576 in one patient treated at 120 mg day^−1^ on day 1 (open symbols) and 4 (closed symbols) of the first cycle.

The plasma concentration–time profiles of XR11576 were similar for all patients studied, with a representative example shown in Figure 2

**Figure 3:**
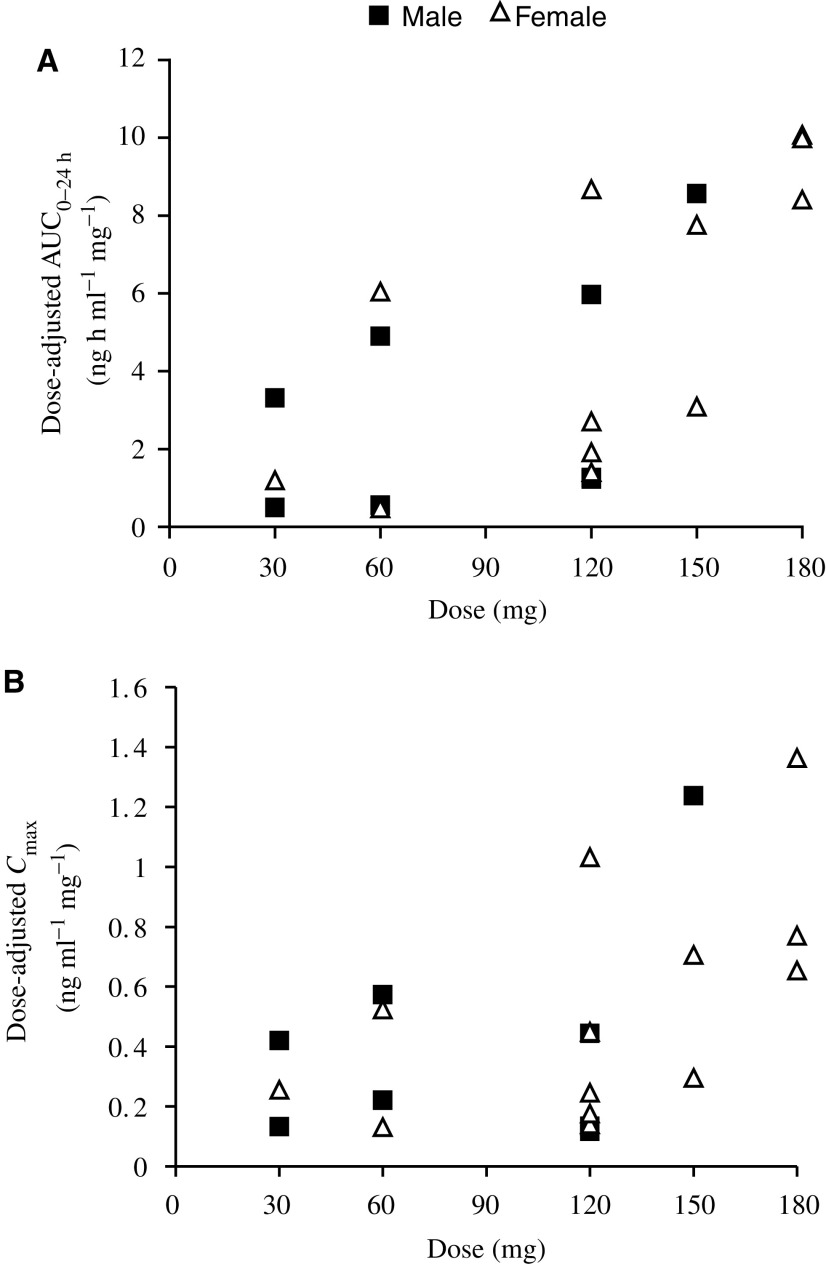
Relationship between AUC (**A**) and *C*_max_ (**B**) on day 1 of the first cycle of XR11576 as a function of dose administered per day.

. Maximum peak drug levels occurred at 3.69±0.79 h post dose and declined with a mean terminal half-life of up to 70 h. Inspection of the scatterplots of dose *vs* either AUC_0–∞_ (Figure 3A

) or *C*_max_ (Figure 3B) for XR11576 revealed an increase of both parameters with the dose level administered.

The long half-life relative to the dosing interval resulted in accumulation in plasma of XR11576, such that exposure was approximately two-fold higher by the last day of dosing.

The intrapatient variability was assessed by comparing the AUC_0–24 h_ values on days 1 and 4 during cycle 1 with the corresponding values during cycle 2. In general, systemic exposure during cycle 1 was not appreciably different from that observed during cycle 2, with average AUC_0–24 h_ ratios ranging from 0.9 to 1.6. The interpatient variability in the observed PKs was large, with coefficients of variation in AUC values as high as >100%.

Over the dose levels studied, the systemic exposure to XR11576 increased with increasing dose. Overall, systemic exposure increased in a greater than dose-proportional manner (Figure 3). The mean PK parameters determined using a noncompartmental analysis are listed in Table 4

**Table 4:** Summary of the pharmacokinetics of XR11576 during the first treatment course

**Dose level (mg day^−1^)**	***N***		**AUC_0-t_ (ng h ml^−1^)**	**AUC_0–∞_ (ng h ml^−1^)**	***C*_max_ (ng ml^−1^)**	***T*_max_ (h)**	***T*_1/2_(h)**	***R*_0_**
30	3							
		Mean	36.2	50.0	8.07	2.5	5.53	1.56
		cv%	83.0	88.2	53.8	34.6	75.4	17.9
60	4							
		Mean	178	517	21.7	3.50	68.8	1.87
		cv%	98.3	26.3	60.5	16.5	70.4	30.2
120	8							
		Mean	329	535	41.1	3.75	31.2	1.93
		cv%	109	94	90.4	27.6	162	78.6
180	3							
		Mean	970	1812	112	4.67	NC	1.34
		cv%	45.7	27.6	63.4	24.7	NC	7.1
150	3							
		Mean	1708	2236	167	4.00	NC	1.53
		cv%	9.89	7.78	40.9	0	NC	5.9

*N*=number of patients; cv=coefficient of variation; AUC=area under the concentration–time curve; *C*_max_=peak plasma level; *T*_max_=time to maximal concentration;*T*_1/2_=terminal elimination half-life; *R*_0_=ratio of accumulation; NC=not calculated as terminal exponential phase could not be unambiguously identified.

**Figure 4:**
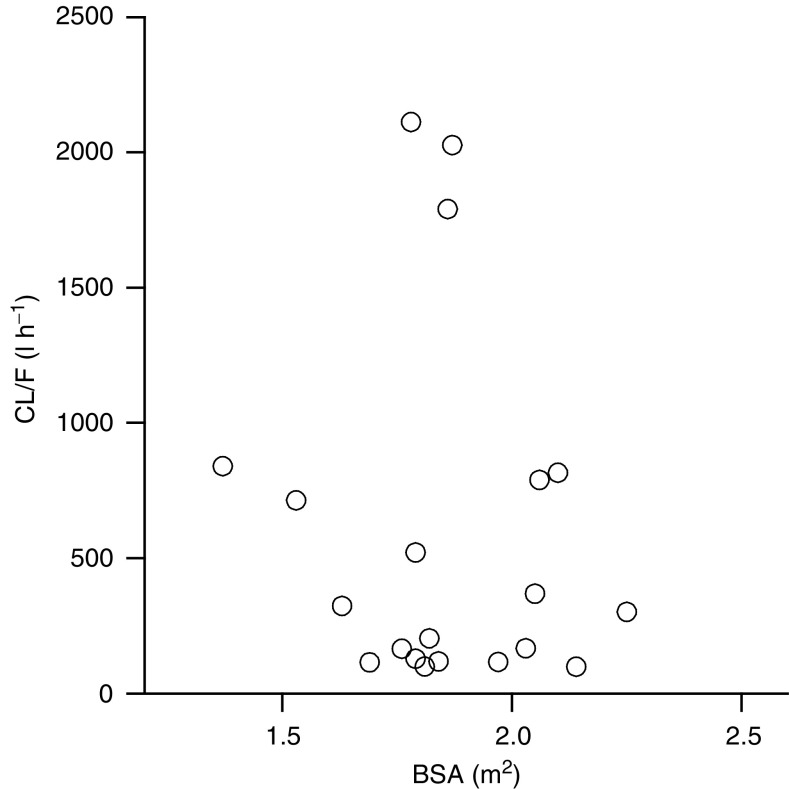
Relationship between the absolute apparent CL/F (calculated by dividing the absolute administered oral dose of XR11576 by the AUC of XR11576).

. In all patients, the PKs of XR11576 were also determined in the second cycle. There were no significant differences between PK parameters derived from paired data sets (data not shown), suggesting a time-independent PK behaviour when XR11576 is administered on days 1–5 every 21 days. Less than 0.5% of the administered dose of XR11576 was excreted unchanged in urine within 24 h after drug administration. To test the appropriateness of flat dosing strategy in this study, the BSA was plotted *vs* the absolute apparent CL/F (calculated by dividing the absolute administered oral dose of XR11576 by the AUC of XR11576) in Figure 4

. As expected, no relationship was found between the apparent CL/F and BSA.

## DISCUSSION

Dual topoisomerase I and II inhibition might have advantages over either topoisomerase I or II inhibition since both cell cycle-dependent and -independent topoisomerases are targeted. In preclinical studies, this property resulted in enhanced antitumour activity. The availability of an oral formulation of XR11576, a dual topoisomerase inhibitor, for clinical use would enable a convenient method of prolonged drug administration and provides the opportunity for cost-effective outpatient therapy. In the present study, XR11576 was administered orally on a daily-times 5 regimen every 3 weeks. Dose-limiting toxicity consisted of diarrhoea and fatigue. Diarrhoea is a well-known side effect of camptothecin and its derivatives. Oral administration of irinotecan (Soepenberg *et al*, 2002), 20-*S*-camptothecin (Natelson *et al*, 1996), 9-nitrocamptothecin (Verschraegen *et al*, 1998), topotecan (Creemers *et al*, 1997; Gerrits *et al*, 1998) and 9-AC (Mani *et al*, 1998; De Jonge *et al*, 1999) induced diarrhoea in 24–54% of administered cycles. Prolonged oral administration (21 days) of topotecan resulted in severe diarrhoea in 22%, which could not be controlled with loperamide (Creemers *et al*, 1997). In our present study, diarrhoea grade 1–2 was observed in 26% and grade 3–4 in 6% of the cycles. In most patients diarrhoea was self-limiting, not requiring any therapy.

Although nausea and vomiting were not considered as a DLT, all patients at the recommended dose level of 120 mg day^−1^ experienced nausea and/or vomiting grade 1–2, despite a vigorous prophylactic antiemetic regimen. With the exception of the first two dose levels, nausea and vomiting started within the first 2 days of treatment and tended to have a more protracted course with increasing dose (median duration 2 days (range 1–5) at 30 mg to a median duration of 6 days (range 1–20) at dose level 180 mg). Acknowledging that this is a major drawback for an oral formulation, an alternative regimen employing day 1 and 8 administration every 21 days is being evaluated with the assumption that such a regimen would require a more limited use of antiemetics. If this schedule results in a higher dose intensity and more manageable gastrointestinal side effects, it will be considered for phase II testing.

Haematological toxicity was mild in this study and not clearly dose- or exposure-related. This is in contrast to the haematological toxicity observed with most topoisomerase I inhibitors. The limited haematological toxicity might be related to the relatively limited systemic exposure to the drug, although the AUC values measured from the dose level of 120 mg onwards were within the range of AUC values associated with preclinical activity.

In the present study, the systemic exposure to XR11576 rose more than proportional to increasing dose. Oral bioavailability studies have not been performed because of lack of an intravenous formulation of the drug. XR11576 was administered at fixed doses during the study. Using linear regression analysis, XR11576 oral clearance was not significantly related to patient body surface area, confirming that the application of dosing per body surface area would not have optimised dosing of this agent.

In this study, the DLTs of XR11576 were diarrhoea and fatigue. The recommended dose for phase II studies of XR11576 is 120 mg administered orally, on days 1–5 every 21 days. Alternative regimens are currently being explored.
